# Thermomechanical Behavior of Bone-Shaped SWCNT/Polyethylene Nanocomposites via Molecular Dynamics

**DOI:** 10.3390/ma14092192

**Published:** 2021-04-24

**Authors:** Georgios I. Giannopoulos, Stylianos K. Georgantzinos

**Affiliations:** 1 Department of Mechanical Engineering, School of Engineering, University of Peloponnese, 1 Megalou Alexandrou Street, GR-26334 Patras, Greece; 2 Department of Aerospace Science and Technology, National and Kapodistrian University of Athens, GR-34400 Psachna Evias, Greece; sgeor@uoa.gr

**Keywords:** bone-shaped, fullerene, nanotube, polymer, nanocomposite, stress-strain

## Abstract

In the present study, the thermomechanical effects of adding a newly proposed nanoparticle within a polymer matrix such as polyethylene are being investigated. The nanoparticle is formed by a typical single-walled carbon nanotube (SWCNT) and two equivalent giant carbon fullerenes that are attached with the nanotube edges through covalent bonds. In this way, a bone-shaped nanofiber is developed that may offer enhanced thermomechanical characteristics when used as a polymer filler, due to each unique shape and chemical nature. The investigation is based on molecular dynamics simulations of the tensile stress–strain response of polymer nanocomposites under a variety of temperatures. The thermomechanical behavior of the bone-shaped nanofiber-reinforced polyethylene is compared with that of an equivalent nanocomposite filled with ordinary capped single-walled carbon nanotubes, in order to reach some coherent fundamental conclusions. The study focuses on the evaluation of some basic, temperature-dependent properties of the nanocomposite reinforced with these innovative bone-shaped allotropes of carbon.

## 1. Introduction

The majority of the nanocomposite (NC) problems and applications are typically related to combinations of loads instead of single types of loads. Perhaps the most common problem is the study of an NC under the simultaneous action of mechanical as well as thermal loadings. Nowadays, the research on nanomaterial reinforced composites that are subjected to thermomechanical loads is of great interest since it may provide valuable practical and efficient solutions in a variety of novel applications. Today, intensive research is carried for the production of polymer-based nanocomposites with special and enhanced thermal conductivity properties for use in thermal management systems [1,2] or, on the contrary, for the development of nanofilled polymers for thermal insulation applications from energy storage to power delivery [3]. Additional attention is paid in the field of structural applications where nanoreinforced polymers seem to be ideal candidates for high-temperature operation devices [4,5]. The accurate prediction of the thermomechanical properties especially of polymer-based NCs, which provide enhanced mechanical characteristics such as high strength-to-weight ratio, is of high importance. In this context, Burgaz [6] has investigated the current status of thermomechanical properties of polymer NCs containing nanofillers in the form of nanocylinders, nanospheres, and nanoplatelets, using case studies from the literature to highlight significant innovations and potential applications. In another interesting attempt, Reddy et al. [7] have discussed some of the recent developments in multiscale modeling of the thermal and mechanical properties of advanced NC systems by including relevant works from the literature to improve the theoretical background. 

Theoretical approximations for analyzing the thermomechanical properties of NCs include molecular dynamics (MD) [8,9,10,11,12,13,14,15,16], molecular mechanics (MM) [17,18], and continuum mechanics (CM) [19] based methods. In addition, multi-scale numerical schemes have recently been proposed, which combine atomistic simulations such as MD or MM with other CM methods such as FEM in an effort to provide reliable predictions with low-computational cost [17,18,20]. Despite the fact that the MM and the CM formulations require significantly smaller computational efforts, the MD approaches seem to be more versatile and provide more accurate and reliable numerical solutions when investigating multiphase nanomaterial components in the nanoscale. This is due to the variety of potential models, force fields, algorithm choices, and simulation modulus that are available in most of the relevant commercial codes [21].

There are several recent attention-grabbing works associated with the MD simulation of the thermomechanical behavior of carbon nanomaterial reinforced polymers. In a relatively early effort, Cho and Yang [8] performed a parametric study to investigate the effects of composition variables on the thermic and mechanical properties of carbon nanotube (CNT)-reinforced NCs using MD simulations. Aiming at a different outcome, Liu et al. [9] adopted classical MD simulations to investigate the absorption and diffusion behavior of polyethylene (PE) chains on the surface of the side-wall of a CNT at different temperatures. Much later, Herasati et al. [10] investigated the effects of polymer chain branches, crystallinity, and CNT additives on the glass transition temperature of PE. In a characteristic attempt, Jeyranpour et al. [11] adopted MD to carry out a comparative study regarding the effects of fullerenes on the thermo-mechanical properties of a specialized resin epoxy. An extended study was performed by Pandey et al. [12] who focused on the study of viscoelastic, thermal, electrical, and mechanical properties of graphite flake-reinforced high-density PE composites. Adopting a different matrix material, Zhou et al. [13] conducted a comparative study to determine the effects of graphene and CNTs on the thermomechanical properties of asphalt binder using MD. On the other hand, Park et al. [14] investigated the thermomechanical characteristics of silica-mineralized nitrogen-doped CNT reinforced poly (methyl methacrylate) (PMMA) NCs for the first time by MD simulations. An interesting study was presented by Singh and Kumar [15] in which they examined the interfacial behavior of functionalized CNT/PE NC at different temperatures using MD simulations, utilizing the second-generation polymer consistent force field (PCFF). Finally, in a more recent attempt, Zhang et al. [16] investigated via MD simulations the thermomechanical properties of a NC consisting of weaved PE and CNT junctions.

At least at the micro-scale, it is well established that bone-shaped (BS) fibers may carry the load more effectively and provide higher fiber pull-out resistance because of the mechanical interlocking between the enlarged fiber ends and the matrix [22]. On the other hand, in a notable attempt, Xu et al. [23] presented a novel approach for the template synthesis of BS CNT nanomaterials. Taking into consideration the aforementioned facts, in the present work, the reinforcing ability of a recently presented nanofiber (NFB) [24], when used as filler in a polymeric matrix made of PE, is numerically analyzed via MD simulations at various temperature levels. Typically, the circular edges of single-walled carbon nanotubes (SWCNTs) are capped with fullerene hemispheres of the same diameter [25]. However, here, the SWCNT edges are enlarged by attaching to them giant spherical molecular formations, which are based on the atomistic structure of stable high-order carbon fullerenes [26]. The NC is tested by using a periodic unit cell that contains at its center this special carbonic single-walled molecular structure as reinforcement. The proposed BS NFB is surrounded by a number of PE chains composing the polymeric matrix phase. A uniform and periodic NFB dispersion is assumed at a rather high mass fraction of 20% in order to better unveil all the temperature-dependent reinforcing effects. The thermomechanical behavior of the NC is examined via the presentations of various temperature-dependent diagrams regarding its axial stiffness coefficients, tensile strength, and linear coefficient of thermal expansion. The influence of the temperature rise on the longitudinal and transverse tensile stress–strain behavior is also illustrated. At all times, for comparison reasons, the BS NFB/PE NC under major investigation is set into contrast with an equivalent PE NC reinforced by an ordinary capped (OC) SWCNT of the same tubular diameter and total length. To the author’s best knowledge, this is the first time that the effects of this BS NBF on the thermomechanical behavior of a polymer are being examined via MD or any other theoretical approach.

## 2. Primary Geometry and Density Assumptions

### 2.1. Structure of Single Molecules

**Figure 1:**
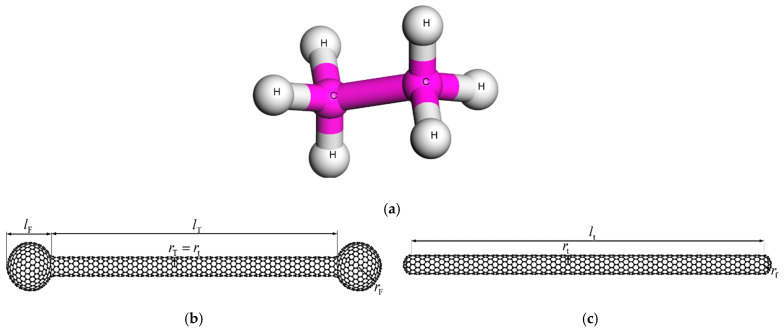
Atomistic structure of the: (**a**) repeat unit of the PE chain, (**b**) BS SWCNT, and (**c**) OC SWCNT.

Typically, the PE matrix phase is assumed to consist of polymeric chains of 100 monomers. The repeat unit of the PE chains is illustrated in Figure 1a. Figure 1b depicts the molecular structure and basic geometric characteristics of the investigated BS SWCNT, while Figure 1c shows the atomistic formation of the OC SWCNT, which is also tested for comparison reasons.

The tubular shape of both NFBs is achieved by using the molecular structure of the zigzag (10,0) SWCNT, the radius of which is *r*_t_ = 0.397 nm [25]. The edges of the BS SWCNT are capped by using enlarged spherical segments based on the molecular structure of C_500_ fullerene [26]. The radius and the length of the spherical C_500_ fullerene-like segment are *r*_F_ = 0.997 nm [26] and *l*_F_ = 1.912 nm < 2 *r*_F_, respectively. The length of the (10,0) SWCNT-like tubular shape is *l*_T_ = 12.58 nm, leading to a total BS NFB length of 16.40 nm. 

On the other hand, the edges of the OC SWCNT are formed by using the hemispherical molecular structure of C_60_ fullerene [25]. The radius of C_60_ hemisphere is obviously equal to *r*_f_ = *r*_t_ = 0.397 nm [25]. By selecting the specific nano-dimensions, the total length of the OC NFB becomes 16.30 nm, which is almost equal to the BS NFB total length. Thus, a comparison of the reinforcing ability between these two types of NFBs may be enabled. 

It may be proved that the lattice area *A*_NFB_ of each NFB is given by:

(1) $$A_{\mathrm{NFB}}\approx \{\begin{array}{ll} 2\pi r_{t}^{}l_{T}+2[(2\pi r_{F}^{2}+2\pi r_{F}^{}(l_{F}^{}-r_{F}^{})], & \mathrm{NFB}=\mathrm{BS}\;\mathrm{SWCNT}\\ 2\pi r_{t}^{}l_{t}+2(2\pi r_{f}^{2}), & \mathrm{NFB}=\mathrm{OC}\;\mathrm{SWCNT} \end{array}$$

The total number of atoms *N*_NFB_ of the BS and OC NFB is 2130 and 1520, respectively. In addition, the wall thickness of both NFBs is assumed to be equal to the usual distance between two successive carbon layers in graphite, i.e., *t* = 0.335 nm. Given the specific wall thickness, the density of each NFB may be approximated by the following equation:

(2) $$\rho_{\mathrm{NFB}}=\frac{m_{\mathrm{NFB}}}{A_{\mathrm{NFB}}t}$$

where *m*_NFB_ is the mass of the NFB, which may easily be calculated by the relationship:

(3) $$m_{\mathrm{NFB}}=N_{\mathrm{NFB}}m_{C}$$

where *m*_C_ = 1.9927 × 10^−23^ g is the mass of a carbon atom.

### 2.2. Unit Cells

**Figure 2:**
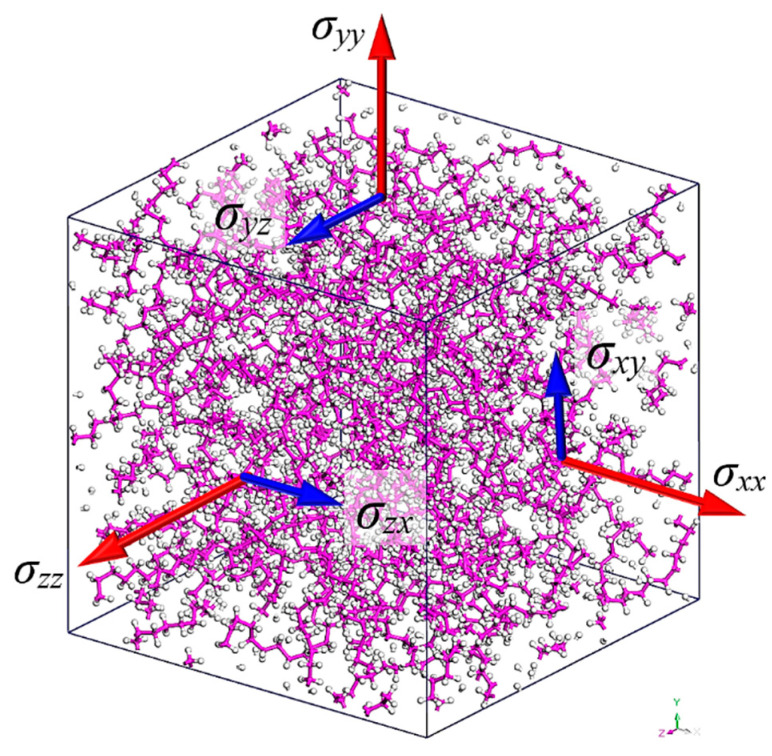
The equilibrated unit cell of the pure PE at *T* = 300 K.

The initial model domains are analyzed according to a global Cartesian coordinate system (*x*,*y*,*z*). In order to comprehensively examine the two-phase NC models, the pure PE amorphous material should be analyzed beforehand for each tested temperature level *T* = 300, 325, 350, 375, 400 K. It should be mentioned that all simulations are performed for temperatures higher than the glass transition temperature of polyethylene [10]. For all cases, it is assumed that the PE has an initial density equal to ^in^*ρ*_PE_ = 0.6 g/cm^3^. According to this PE density value, by utilizing 10 PE polymer chains and by taking into account the molecular weight of each PE chain, a cubic unit cell of equal initial side lengths of ^in^*L*_PE*x*_, ^in^*L*_PE*y*_, and ^in^*L*_PEz_ along the *x*-, *y*-, and *z*-axis is constructed [21]. It should be noticed that the use of more than 10 chains inside the unit cell had a negligible effect on the overall numerical outcome regarding the thermomechanical behavior of PE. After conducting the full MD procedure described in the following section, the final converged values of the PE unit cell density ^fi^*ρ*_PE_(*T*), the side lengths ^fi^*L*_PE*x*_(*T*) = ^fi^*L*_PE*y*_(*T*) = ^fi^*L*_PEz_(*T*), as well as the thermomechanical behavior of the PE at a given temperature *Τ* are estimated. The final equilibrated formation of an amorphous unit cell of the pure PE at 300 K is illustrated in Figure 2. The depicted vectors *σ_xx_*, *σ_yy_*, and *σ_zz_* correspond to the normal stresses in the *x*, *y*, and *z* direction, while the vectors *σ_xy_*, *σ_yz_*, and *σ_zx_* denote the shear stresses in the *x-y*, *y-z,* and *z-x* plane, respectively, required for the thermomechanical characterization of a given unit cell. 

The initial geometry of the NC unit cells is defined in a more complicated manner. First, both the BS and the OC SWCNT of Figure 1a,b, respectively, are kept constantly aligned with the *x*-axis. Moreover, their centroid is maintained at the center of the unit cells at all times. To assure an effective distribution of the reinforcements within the polymeric material, it is assumed that the longitudinal length of the NC unit cell ^in^*L*_NC*x*_(*T*) is six times higher than its transverse lengths ^in^*L*_NC*y*_(*T*) and ^in^*L*_NC*z*_(*T*), i.e., ^in^*L*_NC*x*_(*T*) = 6 × ^in^*L*_NC*y*_(*T*) = 6 × ^in^*L*_NC*z*_(*T*). This aspect ratio is kept stable at all times until the molecular structure of the final equilibrated unit cell is achieved, which means that in the final stage of the analysis there is ^fi^*L*_NC*x*_(*T*) = 6 × ^fi^*L*_NC*y*_(*T*) = 6 × ^fi^*L*_NC*z*_(*T*).

In order to enable packing [21] of the PE chains into each unit cell, an initial NC density ^in^*ρ*_NC_ should be predefined. It is convenient to assume that the density of both NFBs *ρ*_NFB_ is negligibly affected within the temperature range from 300–400 K. Then, the initial density of the NC may be estimated by the following relationship:

(4) ρinNC=mNFB+mPEmNFBρNFB+mPEρinPE

where *m*_PE_ is the mass of the PE inside the unit cell. 

The specific mass of the PE may be estimated via the following equation:

(5) $$m_{\mathrm{PE}}=m_{\mathrm{NFB}}\frac{\left(100-M_{\mathrm{NFB}}\right)}{M_{\mathrm{NFB}}}$$

where *M*_NFB_ is the mass fraction of the NFB taken equal to 20% for all cases under consideration.

Evidently, in Equation (4), the initial density of the polymeric matrix component is taken equal to ^in^*ρ*_PE_ = 0.6 g/cm^3^, i.e., the initially assumed density for the construction of the pure PE unit cell. 

It is easy to prove that the initial longitudinal and transverse lengths of the NC unit cell may be calculated by:

(6) LinNCx=6×LinNCy=6×LinNCz=36(mNFBρNFB+mPEρinPE)3

**Figure 3:**
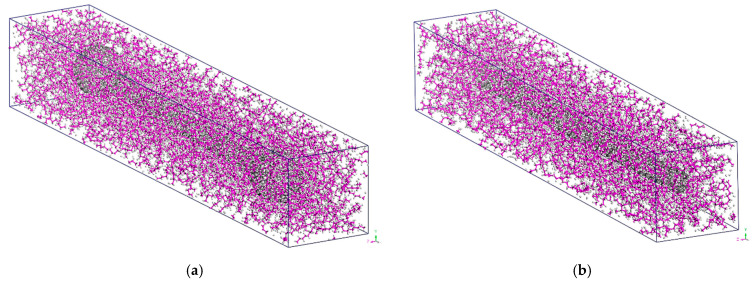
The equilibrated unit cell of the (**a**) BS SWCNT and (**b**) OC SWCNT reinforced PE at a mass fraction of 20% and temperature *T* = 300 K.

Having the initial geometry of the NC fully defined, the MD formulation may be carried out in order to compute the final unit cell shape expressed by the lengths ^fi^*L*_NC*x*_(*T*), ^fi^*L*_NC*y*_(*T*), and ^fi^*L*_NC*z*_(*T*); the density ^fi^*ρ*_NC_(*T*); and the temperature-dependent mechanical behavior characterized by the corresponding stress–strain curves. A representative final equilibrated unit cell of the BS and OC SWCNT reinforced polymer is shown in Figure 3a,b, respectively.

## 3. MD Simulation

The full MD simulation procedure that is proposed here is divided into the following described stages, realized by using the “Materials Studio” software package (Version 2017).

### 3.1. Geometry Optimization of Single Molecular Structures

In the first stage, geometric optimization (GO) [21] is performed for each initially assumed molecular structure, i.e., the main PE chain as well as both NFBs, which are depicted in Figure 1. During the GO, energy minimization is achieved by using the steepest descent algorithm [21]. It is assumed that convergence is accomplished when the absolute difference of the computed system energy and force between two subsequent iterations becomes less than 0.001 Kcal/mol and 5 Kcal/mol/nm, respectively. The required numerical calculations are based on the Dreiding potential that contains four contributing terms for representing bond stretching, changes in bond angle, changes in dihedral rotation, and van der Waals non-bonded interactions. The total energy according to the Dreiding generic force field may be expressed as [27]:

(7) $$\begin{array}{ll} U_{\mathrm{total}} & =\sum_{\mathrm{bond}}\;\left[\frac{1}{2}k_{b}{\left(b-b_{0}\right)}^{2}\right]+\sum_{\mathrm{angle}}\;\left[\frac{1}{2}k_{\theta }{\left(\theta -\theta_{0}\right)}^{2}\right]\\ & +\sum_{\mathrm{dihedral}}\left[\sum_{n=1}^{4}k_{n}{\left(\cos\varphi \right)}^{n-1}\right]+\sum_{\mathrm{nonbond}}\left\{4\varepsilon_{0}\left[{\left(\frac{\delta }{r_{ij}}\right)}^{12}-{\left(\frac{\delta }{r_{ij}}\right)}^{6}\right]\right\} \end{array}$$

In the last equation, the first three sums denote the energies required to stretch bonds from their equilibrium length *b*_0_ to *b*, change bend angles from their equilibrium value *θ*_0_ to *θ*, and twist atoms about their bond axis by an angle *φ*. The final sum, which contains functions of the atom pair distance *r_ij_* denotes the Lennard-Jones-based van der Waals (vdW) non-bond interactions. The constant *ε*_0_ and *δ* is the energy well depth and the zero-energy spacing of the Lennard-Jones potential, respectively. Depending on the atom type combinations, the Dreiding force-field predefines the stiffness-like parameters *k_b_*, *k_θ_*, and *k_n_* [21,27]. It should be mentioned that, here, the vdW contributions are computed according to the atom-based summation method using a cut-off radius of 1 nm and long-range corrections [28].

**Figure 4:**
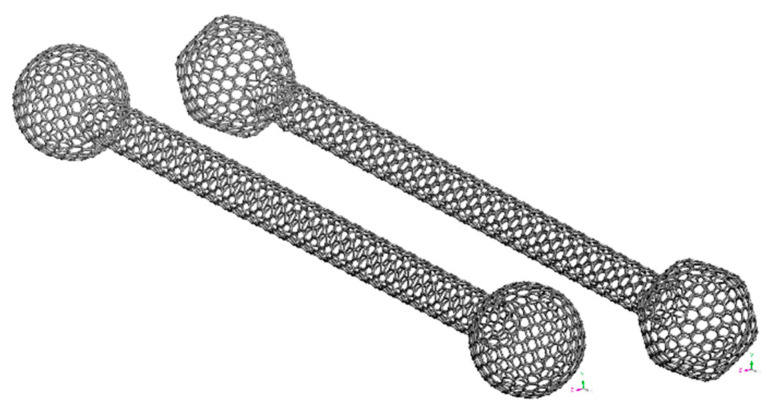
The BS SWCNT molecular structure before and after the GO with respect to the global Cartesian coordinate system (*x*,*y*,*z*).

After conducting the GO of the single BS SWCNT, some negligible cross-sectional asymmetries are revealed on the molecular structure of its edges. Characteristically, Figure 4 demonstrates the molecular configuration of the BS SWCNT after being geometrically optimized in the first stage of the MD analysis. Overall, it may be observed that the enlarged spherical edges obtain a 3d hexagon-like shape which, however, does not influence that provided by the NFB mechanical interlocking phenomena.

### 3.2. Construction and Geometry Optimization of Unit Cells 

In the second stage, the periodic unit cell representing the problem under consideration is constructed using standard packing algorithms available by commercial software packages [21] and by following the procedure that is analytically described in the previous section. After defining the three-dimensional (3d) unit cell box for the tested temperature T, a number of PE chains are inserted into it while the packing algorithm evenly increases their population until the initial unit cell density is achieved, i.e., ^in^*ρ*_PE_ = 0.6 g/cm^3^ or ^in^*ρ*_NC_ of Equation (4) when the pure matrix or the NC is to be analyzed, respectively. Evidently, the relevant positioning of the molecules is performed after computing the interactions between neighbor atoms via the Dreiding force field whereas the single-chain conformations, ring spearing, and close contacts are constantly monitored. To achieve a minimized initial unit cell state, low energy sites are preferred over high energy sites for each molecular structure. A GO process, like the one described in the first stage, is executed to additionally reduce the overall potential energy of the 3d problem domain.

### 3.3. Dynamic Analysis of Unit Cells 

In the third stage, a three-phase dynamic analysis is performed for each investigated temperature *T* by using a time step of 1 fs in all cases. The followed MD numerical scheme including the utilized force field, the equilibrium algorithms, and the dynamic analysis computational process, is similar to the one proposed and validated by Bao et al. [29], in an effort to investigate amorphous PE under cyclic tensile-compressive loading below the glass transition temperature. Due to the dynamic nature of the simulation, in order to keep the molecular systems under a specific temperature and pressure level, the Andersen thermostat and Berendsen barostat are utilized, respectively [28]. Initially, an MD simulation takes place for a 50 ps time period under the NVT ensemble, assuming a temperature of 500 K. Then, the NPT ensemble is utilized for a 250 ps time interval to keep the temperature and the external pressure of the unit cell at 500 K and 1 atm, respectively. Finally, an NPT dynamic analysis is carried out to drop slowly the temperature from 500 K to *T* = 300, 325, 350, 375, and 400 K by adopting 2 ps simulation time per 1 K of temperature decrease. After the finalization of the procedure, the relaxed equilibrium state, true final density, and side lengths of the unit cell are obtained. Performing an NPT dynamic analysis using even higher time intervals or a time step lower than 1 fs had no observable effect on the final numerical solutions. 

### 3.4. Thermomechanical Properties Calculation 

In the fourth and final stage of the process, the tensile stress–strain curves at a given temperature T are computed by applying to the 3d unit cell a set of quasi-static uniaxial tension and shear deformations. To avoid an extraordinary computational cost, the maximum chosen amplitude of strains is +0.1. The normal stresses, i.e., *σ_xx_*, *σ_yy_*, and *σ_zz_*, and shear stresses, i.e., *σ_xy_*, *σ_yz_*, and *σ_zx_*, (see Figure 2) at each strain level may be estimated through the following average virial stress of a system of particles [28]:

(8) $$\bar{\sigma }=\frac{1}{2V}\sum_{j(\neq i)}(m_{ij}⊗u_{ij}+r_{ij}⊗f_{ij})$$

where *V* is the volume of the system, *i* and *j* denote two particles at positions **r***_i_* and **r***_j_*, respectively, **r***_ij_* is equal to **r***_i_*–**r***_j_*, **f***_ij_* is the inter-particle force applied on particle *i* by particle *i*, and **m***_ij_* and **u***_ij_* are the corresponding mass and velocity contributions. 

In order to estimate the axial stiffness coefficients *E_xx_*, *E_yy_*, and *E_zz_*, Hooke’s law may be utilized in the three axial directions as:

(9) $$\sigma_{xx}=E_{xx}\varepsilon_{xx};\;\sigma_{yy}=E_{yy}\varepsilon_{yy};\;\sigma_{zz}=E_{zz}\varepsilon_{zz}$$

where *σ_xx_*, *σ_yy_*, and *σ_zz_* and *ε_xx_*, *ε_yy_*, and *ε_zz_* are the tensional normal stresses and strains in the *x*-, *y*-, and *z*-axis, respectively.

Note that despite the amorphous nanostructure of the pure PE unit cell, it may be assumed that it has an almost isotropic behavior due to the high length accompanied by the random distribution of the polymer chains in the simulation box. Contrary, regarding the NC unit cells, significant anisotropy is present due to the NFB reinforcements. 

Finally, note that the computation of the final unit cell size at the reference temperature *T*_0_ and an arbitrary temperature *T*_1_, permits the calculation of the coefficient of linear thermal expansion *a_L_*. The specific thermal coefficient along the *x*-axis, with which the NFBs are aligned, can be approximated for the temperature range [*T*_0_,*T*_1_] via the following equation:

(10) aLx=LfiUCx(T1)−LfiUCx(T0)T1−T01LfiUCx(T0)

where ^fi^*L*_UC*x*_ is the final unit cell length along the *x*-axis, defined after the MD process is finalized.

## 4. Results and Discussion

For all the material cases under investigation, i.e., the BS SWCNT reinforced PE, the OC SWCNT reinforced PE, and, last but not least, the pure PE, simulations are conducted for five different temperatures, i.e., *T* = 300, 325, 350, 375, and 400 K. Furthermore, all the numerical tests regarding the NC materials correspond to a reinforcement (BS SWCNT of OC SWCNT) mass fraction *M*_NFB_ of 0.2 (20%). Finally, in order to reduce the computational cost without, however, excluding any key information from the numerical solutions, the maximum applied tensile strain is 10% for all cases.

**Figure 5:**
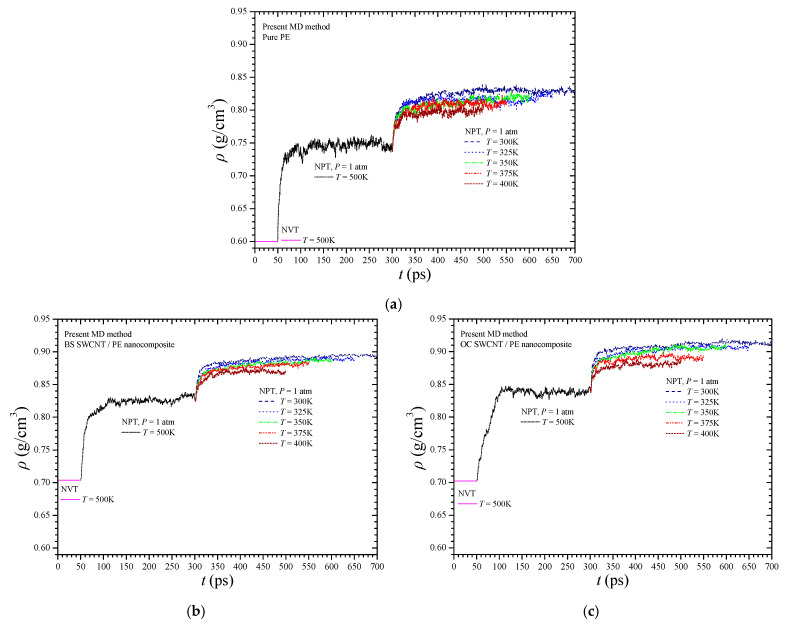
The density of the simulated (**a**) pure PE, (**b**) BS SWCNT-reinforced PE, and (**c**) OC SWCNT-reinforced PE versus time during the dynamic analysis and at various temperatures.

To show the density variations of the BS SWCNT/PE NC, the OC SWCNT/PE NC, and the pure PE during the three-phase dynamic analysis, Figure 5a–c are given, respectively. Each figure shows the density variation of the corresponding unit cell with time, at all the tested temperatures. Generally, the analysis shows that convergence of the density values is achieved after a total MD simulation time of 450 ps. The final density values ^fi^*ρ*(*Τ*), obtained after the three-phase dynamic analysis, correspond to the final points of the curves. These right endpoints of the curves in Figure 5, which define the final converged density at each temperature level, show that the pure PE density values, as expected, are lower than those of the NFB/PE NCs due to the absence of interphase interactions. Note that the use of larger unit cells, i.e., lower mass fractions, would not be so helpful in revealing the effects of the investigated BS NFB and, thus, is avoided here.

**Figure 6:**
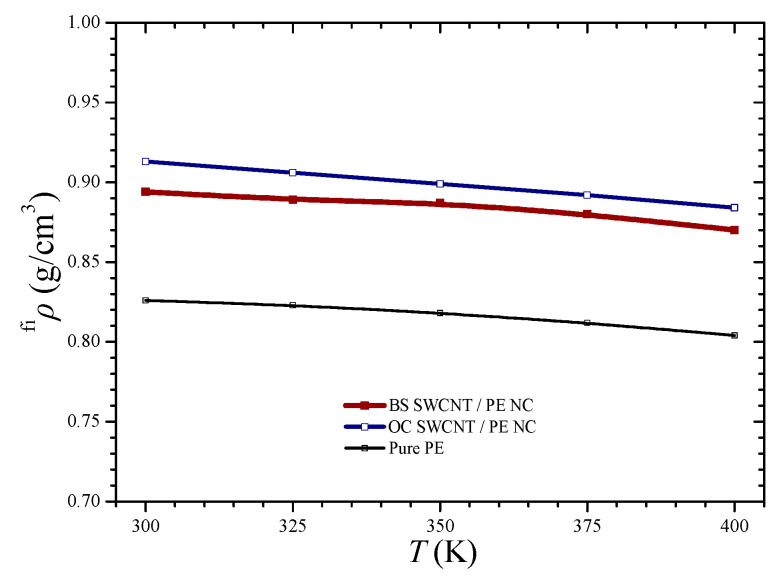
The final density of the simulated materials versus the temperature.

Figure 6 depicts the numerically computed final densities for all the tested materials and temperatures. The density of the pure PE varies between 0.83–0.80 as the temperature increases from 300 to 400 K, a prediction that is in good agreement with other computational and experimental estimations [30,31]. As it can be seen, all the density-temperature variations present an almost linear drop as the temperature increases. The OC SWCNT/PE NC seems to have slightly higher final density values in comparison with the BS SWCNT/PE NC at all temperature levels. However, the linear density decrease of both NC materials presents almost the same slope of decrease. Possibly, the lower density of the BS SWCNT reinforced PE is due to the larger lattice area that the BS NFB presents. The larger the NFB external area, the greater the interface region between the NFB and the matrix, which is characterized by the interlayer distance *t* = 0.335 nm. 

**Figure 7:**
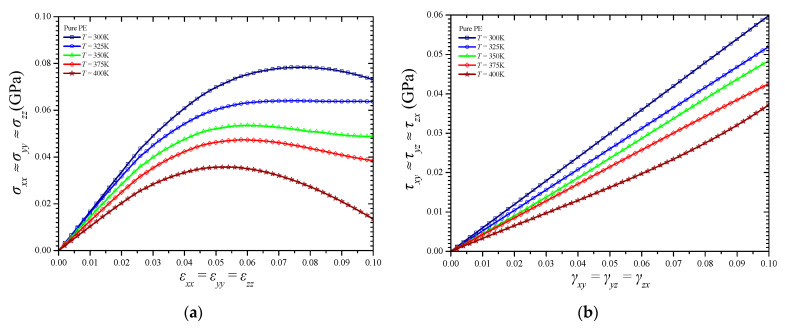
Stress–strain curves in (**a**) tension and (**b**) shear of the pure PE for various temperatures.

Figure 7a,b illustrates the tensile and shear stress–strain behavior, respectively, of the pure amorphous PE material at various temperature levels. Note that the thermomechanical behavior of the PE, in the absence of an NFB reinforcement, is practically isotropic, and thus the same tensile and shear curve applies to all directions. The first peak in the tensile stress–strain curves corresponds to the tensile yield stress of the material. The tensile curve is characterized by a stress-softening region after the yield point. For even higher tensile deformations, PE presents a stress-hardening response [32,33] which, however, may not be illustrated for the rather small strains up to 10% that are investigated here. The tensile yield stress found here for *T* = 300 K is about 78.4 MPa and is in good agreement with the corresponding value of about 76.8 MPa found in an MD computational study based on Dreiding potential model [32]. A higher value of about 108.6 MPa is reported for a room temperature in another similar MD simulation [33]. In addition, an elastic modulus of 1.63 GPa and a Poisson’s ratio of 0.37 are estimated here for the pure PE at 300 K, which are rather lower than the corresponding computed values of 1.32 GPa and 0.32, respectively, found elsewhere [33]. In another MD formulation in which the COMPASS force field has been used instead [34], an elastic modulus of 1.22 GPa and a Poisson’s ratio of 0.37 have been proposed for a temperature of 298 K. For all the tested temperatures, the computed shear stress–strain curves are almost linear for stains up to 10% while their slop decreases in a linear manner as the temperature increases. 

**Figure 8:**
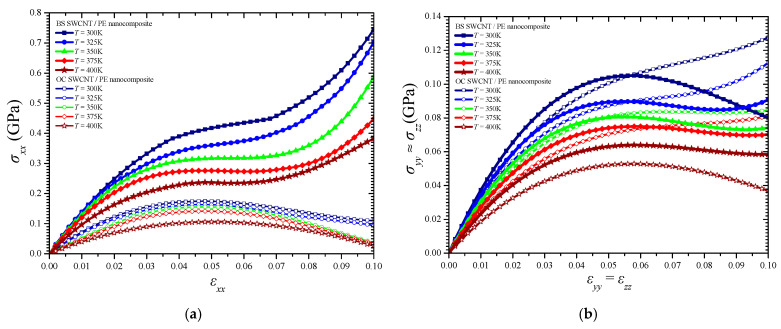
The tensile stress–strain curves of the simulated NCs in the (**a**) longitudinal *x*- and (**b**) transverse *y*- or *z*-axis for various temperatures.

**Figure 9:**
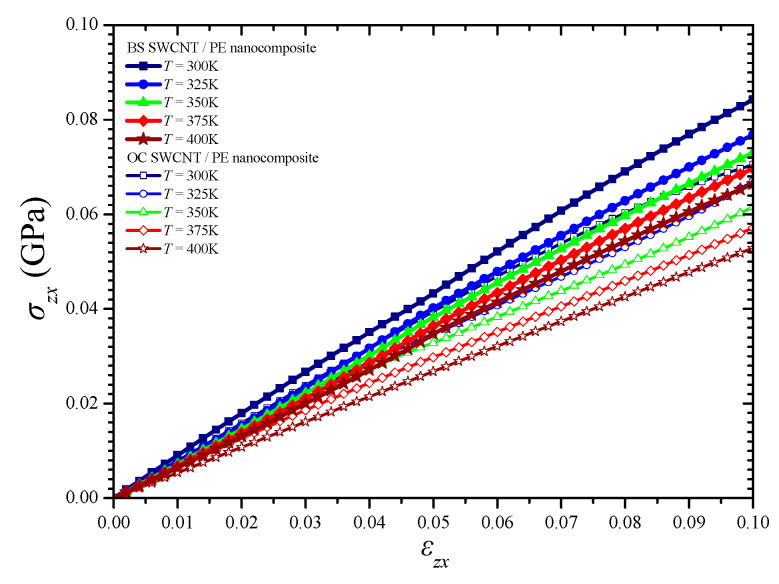
Shear stress–strain curves of the simulated NCs in the *z*-*x* plane for various temperatures.

On the other hand, the computations showed that the NC materials present a distinct behavior along the longitudinal, i.e., effective, direction *x* in which the NFB is oriented, while they demonstrate an almost identical tensional response along the two transverse directions *y* and *z* because of the transverse cross-sectional symmetry of both NFBs. The tensile stress–strain temperature-dependent response of the BS and the OC SWCNT reinforced PE along the longitudinal *x*-axis and transverse *y*- or *z*-axis is illustrated in Figure 8a,b, respectively. In addition, Figure 9 depicts the variation of the *zx* shear stress versus the *zx* shear strain and temperature.

The longitudinal direction *x* presents an upgraded temperature-dependent mechanical response compared with the other two directions. Both NFBs significantly improve the effective mechanical characteristics in the whole temperature range. However, it becomes obvious that the BS SWCNT/PE NC may carry a significantly higher maximum stress than the OC SWCNT/PE one, while it presents an advanced axial stiffness coefficient (tangent of the slope of the linear part of the tensile curves) in the longitudinal direction. This is due to the advanced geometric interlocking and load transfer mechanisms provided by the BS NFB mainly in the longitudinal direction. In addition, the special edge shape of the BS NFB seems to lead to an intense tensile stress hardening in the *x*-axis soon after the yield point is reached. Instead, there is no notable thermomechanical behavior enhancement provided by the BS SWCNT over the OC one, regarding the tensional yield stress in the transverse directions *y* and *z*. Furthermore, almost the same transverse elastic moduli increase may be observed by using both fibers in the PE matrix. Finally, according to the shear stresses–strain variations depicted in Figure 9, the BS NFB seems to offer a rather improved shear stiffness in the *z*-*x* plane for the whole temperature range under investigation. 

**Figure 10:**
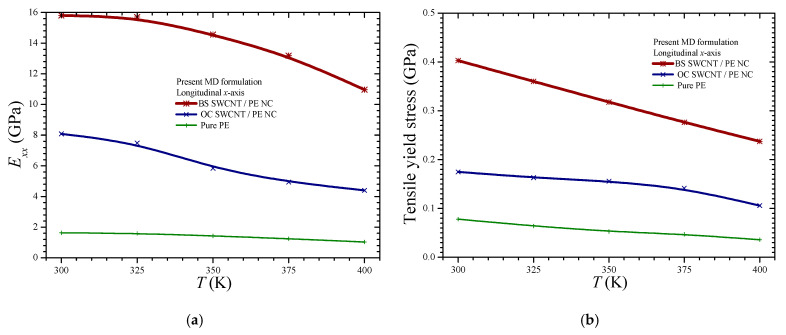
The temperature dependence of the longitudinal (**a**) stiffness coefficient and (**b**) tensile yield stress provided by the three materials.

To better demonstrate the enhancing ability of the BS proposed NFB, the key temperature-dependent axial properties arisen from Figure 7a and Figure 8a are summarized and better analyzed in Figure 10. 

Specifically, Figure 10a,b presents the effective axial stiffness coefficients and the tensile yield stresses of the three investigated materials, i.e., the pure PE, the BS, and OC SWCNT/PE NC, at a variety of temperature levels, respectively. A steady drop in the mechanical performance of all the materials as the temperature rises is observed in both figures. The positive influence of the BS NFB, due to the better 3d interlocking and stress transfer that is provided by its enlarged edges, on both the elastic and yield region, may be concluded by Figure 10a,b, respectively. Figure 10a proves that the BS NFB improves the longitudinal stiffness of the PE more effectively than the OC NFB in the whole investigated temperature range. A significantly higher longitudinal tensile yield stress is observed when the BS SWCNT is used as a reinforcing agent. Specifically, Figure 10b reveals that the BS SWCNT/PE NC may carry at least two times higher axial load than the OC SWCNT/PE NC independently of the temperature level. 

The present numerical tests are carried out considering several simplifications regarding the NFBs such as uniform dispersion, perfect alignment, single-walled molecular structure, straight shape, and specific type and length for the CNT reinforcement. Thus, straightforward comparisons between the present results and corresponding experimental ones using the same NC design parameters may not be provided. Therefore, only a qualitative comparison is attempted with an experimental measurement regarding the elastic modulus of high-density PE (HDPE) reinforced with multi-walled CNTs (MWCNTs), having lengths of 10–30 μm and diameters of 5–15 nm, at a 10% volume fraction [35]. The specific experimentally tested volume fraction is comparable with the one investigated here. It should be mentioned at this point that by using the computed density of the pure PE at 300 K presented in Figure 5a and combining Equations (3) and (5), it may easily be proved that the OC SWCNT mass fraction of 20% corresponds to a volume fraction of about 8.6%. The reported experimental elastic modulus value at room temperature for the above-described MWCNT/HDPE NC is 7.86 GPa [35], which is in good agreement with the present numerical prediction of 8.09 GPa concerning the OC SWCNT/PE NC case.

Finally, the computed values of the linear coefficient of thermal expansion *a_Lx_* along the *x*-axis for the three investigated materials are included in Table 1. The relevant average calculations for the three materials are based on Equation (10). For all cases, a linear increase of the longitudinal length of the unit cells is observed in the temperature range from 300–400 K. The computed linear coefficient of thermal expansion for the pure PE is in excellent agreement with corresponding reported experimental values that typically vary from 1.2 × 10^−4^ to 1.5 × 10^−4^ (1/K) [36]. According to the computed data, the linear coefficient of thermal expansion of the pure PE exhibits a notable increase when filled with both NFBs. However, the influence of the OC SWCNT is more significant, perhaps due to the fact that it leads to denser NC unit cell structures (Figure 6).

Evidently, the extent of accuracy of the presented results is rather affected by the adopted theoretical assumptions as well as the inherent numerical restrictions of the adopted atomistic technique. For example, in the present work, the possible cross-linking phenomena are not considered while a rather high NC mass fraction is investigated in order to minimize the unit cell size and the complexity of molecular interactions and, thus, advance the convergence and the overall computational process. In addition, since the effect of the reinforcement on the NC material yielding becomes apparent by just applying strains up to 10%, the investigation of very high strains up to fracture is avoided. In this way, the computational cost is simultaneously reduced. In addition, the molecular simulations and their outcome are strongly dependent on the adopted potential field, cut-off distance, size of time-step, and equilibrium/convergence criteria. Nevertheless, despite the abovementioned limitations, the reliability of the research conclusions demonstrating the superiority of the proposed BS over the OC NFB, may be considered certain, given that the comparison between the two different reinforcements is realized by using the same theoretical fundamentals and computational options for the whole temperature range under investigation.

## 5. Conclusions

The present study has focused on the numerical prediction of the load-carrying capability of BS NBFs when utilized as reinforcements in polyethylene, under a variety of temperature levels. The computations have been grounded on the MD technique utilizing the Dreiding force field. The molecular structure of the proposed BS NFB has been based on the combination of a typical SWCNT and two giant carbonic fullerenes appropriately attached at the open SWCNT edges. An OC SWCNT of equivalent length and tubular diameter has been also tested in order to reach some distinct conclusions about the superiority of the BS NFB reinforcement regarding the provided temperature-dependent mechanical interlocking at the interphase. The numerical results and comparisons with the standard SWCNT NFB have shown that the proposed NFB increases more considerably both the axial stiffness as well as the tensile yield stress of the NC, especially in the longitudinal direction of the fiber for all the tested temperatures. In the near future, relevant research and relevant MD simulations at temperatures around the glass transition point of the polymeric matrix phase may reveal even more special features of the proposed BS carbonic nanomaterial. 

## Figures and Tables

**Table 1:** The computed values of the linear coefficient of thermal expansion *a_Lx_* for the three materials and the temperature range from 300–400 K.

Simulated Material	Average Computed Linear Coefficient of Thermal Expansion for *x*-axis *a_Lx_* (1/K)
Pure PE	1.431 × 10^−4^
OC SWCNT/PE nanocomposite	1.257 × 10^−4^
BS SWCNT/PE nanocomposite	1.056 × 10^−4^

## Data Availability

The data presented in this study are available on request from the corresponding author.
